# Role of Posterior Tibial Slope Osteotomy in Revision Anterior Cruciate Ligament Reconstruction: A Narrative Review

**DOI:** 10.7759/cureus.104826

**Published:** 2026-03-07

**Authors:** Javier Gonzalez Almonacid

**Affiliations:** 1 Orthopedics, Hospital Clínico San Borja Arriarán, Santiago, CHL; 2 Orthopedics, Clínica Alemana de Santiago, Santiago, CHL

**Keywords:** anterior cruciate ligament, knee biomechanics, posterior tibial slope, revision acl reconstruction, slope-reducing osteotomy

## Abstract

Failure after anterior cruciate ligament (ACL) reconstruction remains a significant clinical challenge, particularly in the revision setting. Among biomechanical factors, increased posterior tibial slope (PTS) is an important factor to consider during the preoperative evaluation of patients undergoing revision ACL reconstruction.

A narrative review of the current literature was performed to analyze the biomechanical and clinical role of PTS in ACL graft failure. Emphasis was placed on revision ACL reconstruction, including the biomechanical rationale for slope correction, proposed threshold values, surgical indications, and reported clinical outcomes of slope-reducing osteotomy. A representative clinical case is presented to illustrate the application of these concepts in practice.

Biomechanical studies consistently demonstrate that increased PTS leads to higher anterior tibial translation and increased ACL graft forces under axial loading. Clinical evidence supports an association between pathological PTS, most commonly values ≥12°, and increased rates of ACL graft failure in both primary and revision reconstructions. In selected revision cases, slope-reducing proximal tibial osteotomy has been shown to decrease graft loading, improve knee stability, and reduce the risk of recurrent failure. However, available clinical data remain limited and are primarily derived from case series and cohort studies.

PTS represents a clinically relevant and potentially modifiable risk factor in revision ACL reconstruction. Slope-reducing osteotomy should be considered as part of a targeted revision strategy in carefully selected patients with pathological PTS, particularly when values exceed 12°. This procedure should not be applied routinely, but rather integrated into a comprehensive revision approach based on individual biomechanical and clinical factors.

## Introduction and background

Anterior cruciate ligament (ACL) reconstruction is a common surgical procedure in orthopedic practice, reflecting the high incidence of ACL injuries worldwide. The reported incidence of ACL rupture ranges between approximately 30 and 78 per 100,000 person-years, depending on age, sex, and activity level [1,2].

Despite advances in surgical techniques and rehabilitation protocols, ACL reconstruction failure remains a relevant clinical problem. Reported failure rates in the literature vary, but generally range between 2% and 6%, with higher rates observed in young, active populations and in high-demand athletes [3-5].

Several risk factors have been associated with graft failure and should be systematically evaluated in patients undergoing revision ACL reconstruction, as ACL graft failure is widely recognized as a multifactorial process. These include technical factors such as tunnel malposition, biological factors related to graft incorporation, and biomechanical factors, including an increased posterior tibial slope (PTS) and a narrow intercondylar notch [6-8].

Identification and correction of modifiable risk factors are essential to reduce the risk of subsequent graft failure in revision settings [3,8]. Among these factors, PTS has gained increasing attention due to its biomechanical effect on anterior tibial translation and increased graft loading and is increasingly recognized as a relevant risk factor for ACL graft failure, particularly in the revision setting [7,9,10].

The purpose of this narrative review is to analyze the role of PTS as a risk factor for ACL graft rupture, to discuss current indications for slope-correcting osteotomy in revision ACL reconstruction, and to summarize the expected clinical outcomes reported in the literature.

## Review

Causes of ACL reconstruction failure 

The causes of ACL reconstruction failure can be broadly classified into technical, biological, and biomechanical factors [7,11].

Technical factors represent the most frequently reported causes of failure, with tunnel malposition consistently identified as the leading contributor. Several authors have suggested that incorrect femoral and/or tibial tunnel placement may account for up to 60-70% of ACL reconstruction failures [12,13]. Graft selection also plays a relevant role, particularly the use of allografts in young and high-demand patients, which has been associated with higher failure rates compared with autografts [4,14].

Biological factors are primarily related to graft incorporation and ligamentization, processes that may vary significantly between patients and can be influenced by individual healing capacity and local biological conditions [15,16].

Biomechanical factors include high-grade pivot shift, residual knee laxity, and altered lower limb alignment. Among these, increased PTS has received particular attention due to its effect on anterior tibial translation and graft loading [6,7,9,10].

Failure to adequately identify and address these factors during preoperative evaluation and surgical planning may result in suboptimal postoperative outcomes and an increased risk of graft failure, especially in the revision setting [3,7,8].

PTS and graft loading in ACL revision

PTS is defined as the angle between the mechanical axis of the tibia in the sagittal plane and the inclination of the tibial plateau, most commonly measured at the medial plateau on lateral radiographs [7]. Although different imaging modalities and reference axes have been proposed, lateral knee radiographs including at least 15 cm of proximal tibia are considered acceptable for clinical assessment, particularly in the revision setting [7].

An increased PTS alters sagittal knee biomechanics by generating an anteriorly directed shear force on the tibia during axial loading. As the ACL is the primary restraint to anterior tibial translation, variations in PTS have a direct impact on native and reconstructed ACL strain [9]. Giffin et al. showed that progressive increases in tibial slope resulted in a proportional increase in anterior tibial translation and ACL graft forces in cadaveric knee models [9].

This biomechanical relationship is particularly relevant during weight-bearing activities, where compressive forces across the knee joint are transformed into anterior shear forces proportional to the magnitude of the posterior slope. As a result, the reconstructed ACL is subjected to higher in situ forces, even in the absence of rotational instability [9,17].

Importantly, slope-reducing osteotomy has been shown to significantly decrease ACL graft forces under axial loading conditions, supporting the concept that PTS is a modifiable biomechanical risk factor [17].

Cadaveric and biomechanical studies provide strong experimental support for the role of PTS in ACL graft loading. Imhoff et al. demonstrated that anterior closing-wedge slope-reducing osteotomy significantly reduced anterior tibial translation under axial load and decreased ACL graft forces in reconstructed knees in cadaveric models [17].

In their study, slope reduction led to a relative decrease of up to 33% in graft forces under higher axial loads, highlighting the protective effect of slope correction on the reconstructed ligament, particularly in high-risk scenarios such as revision ACL reconstruction [17].

These findings reinforce the concept that addressing sagittal alignment can substantially influence graft biomechanics beyond tunnel placement or graft choice alone.

Clinical studies have consistently identified increased PTS as a significant risk factor for ACL graft rupture. Long-term follow-up studies have demonstrated a strong association between high PTS and repeat ACL injury, particularly in young and active populations. Salmon et al. reported that patients with a PTS ≥12° had dramatically reduced graft survival at 20 years, a phenomenon described as the "catastrophic effect" of slope [18].

More recent evidence suggests that PTS remains a dominant risk factor even when adjunctive procedures are performed. Mazy et al. showed that despite the addition of lateral extra-articular tenodesis, patients with a PTS ≥12° experienced significantly higher graft rupture rates compared with those with lower slopes [19].

Furthermore, increased static anterior tibial translation, closely related to PTS, has been identified as an independent predictor of graft failure, emphasizing the importance of sagittal plane biomechanics in ACL reconstruction outcomes [19].

Although a universally accepted pathological threshold for PTS has not been established, values ≥12° are most frequently associated with increased risk of graft failure in both primary and revision ACL reconstruction [7,18,19].

An international expert Delphi consensus highlighted the absence of a single absolute cutoff value for surgical correction but emphasized individualized decision-making based on PTS magnitude, clinical instability, and history of graft failure [7]. Most experts agree that failure to address markedly increased PTS in selected patients may contribute to recurrent graft failure despite technically adequate revision surgery.

Increased PTS should be considered an independent and clinically relevant risk factor for ACL graft failure. Higher slope values lead to increased anterior tibial translation and graft loading, particularly under axial load. In revision ACL reconstruction, failure to recognize and address excessive PTS may explain repeated graft failures, even when other technical factors have been corrected [7,9,17].

Rationale for slope-reducing (deflection) osteotomy in revision ACL reconstruction

Slope-reducing osteotomy has emerged as a valuable surgical strategy in selected patients undergoing revision ACL reconstruction with increased PTS. As previously discussed, increased PTS results in higher anterior tibial translation and increased strain on the ACL graft under axial loading conditions, which may contribute to graft failure despite technically adequate reconstruction [7,9,17].

In the revision setting, where multiple technical factors may already have been addressed, persistent biomechanical overload related to sagittal alignment represents a potentially modifiable risk factor. Slope-reducing osteotomy aims to decrease anterior shear forces across the knee by altering tibial geometry, thereby reducing graft loading and improving the biomechanical environment for graft survival [17].

Indications for slope-reducing osteotomy in revision ACL reconstruction 

There are no absolute indications for slope-reducing osteotomy, and increased PTS represents the fundamental prerequisite for considering this procedure. Current evidence and expert consensus suggest that slope-reducing osteotomy should be considered in selected patients with pathological PTS, most commonly ≥12°, particularly in the revision setting [7,18,19].

Additional clinical factors may strengthen the indication when present in combination with increased PTS, including the following: recurrent ACL graft failure despite appropriate tunnel placement and graft selection, persistent anterior tibial translation, particularly when associated with increased static anterior tibial translation, and high-grade residual instability in young, high-demand patients. Importantly, these factors do not constitute indications for slope-reducing osteotomy in isolation, but rather serve as contextual elements that support surgical decision-making when excessive PTS is identified as a relevant contributor to graft failure [7,17].

An international expert Delphi consensus emphasized that slope-reducing osteotomy should not be performed systematically, but should be reserved for carefully selected cases in which increased PTS is believed to play a significant role in graft failure and persistent instability [7].

Potential contraindications include advanced tibiofemoral osteoarthritis, limited knee range of motion, and cases in which PTS is within physiological limits and graft failure can be explained by other factors such as tunnel malposition or technical errors [17].

Surgical techniques and strategies

Several surgical techniques for slope reduction have been described, with anterior closing-wedge proximal tibial osteotomy being the most commonly reported. This procedure can be performed either above (supratuberositary) or below (infratuberositary) the tibial tubercle depending on the surgical technique and desired correction. The technique involves the removal of an anterior wedge of bone from the proximal tibia, followed by controlled closure to reduce the posterior slope while preserving coronal alignment [17].

Careful preoperative planning is essential to determine the magnitude of slope correction required and to avoid overcorrection, which may result in increased posterior cruciate ligament strain or altered knee kinematics. Most authors aim to reduce the PTS to a physiological range, typically between 7° and 9°, although the optimal target may vary depending on individual anatomy and clinical context. Fixation is typically achieved using locking plates, providing stable fixation [7].

Slope-reducing osteotomy may be performed as a staged procedure or concomitantly with revision ACL reconstruction, depending on tunnel position, graft choice, and surgeon preference. While single-stage procedures have been reported, many authors advocate a staged approach in complex revision cases to optimize tunnel placement and biological conditions [7,17].

Clinical case illustration

An 18-year-old male patient sustained a primary ACL rupture and underwent ACL reconstruction. Nine months after surgery, he experienced a traumatic fall after jumping, involving torsional forces and valgus stress to the operated knee. Subsequent magnetic resonance imaging confirmed rupture of the ACL graft.

**Figure 1:**
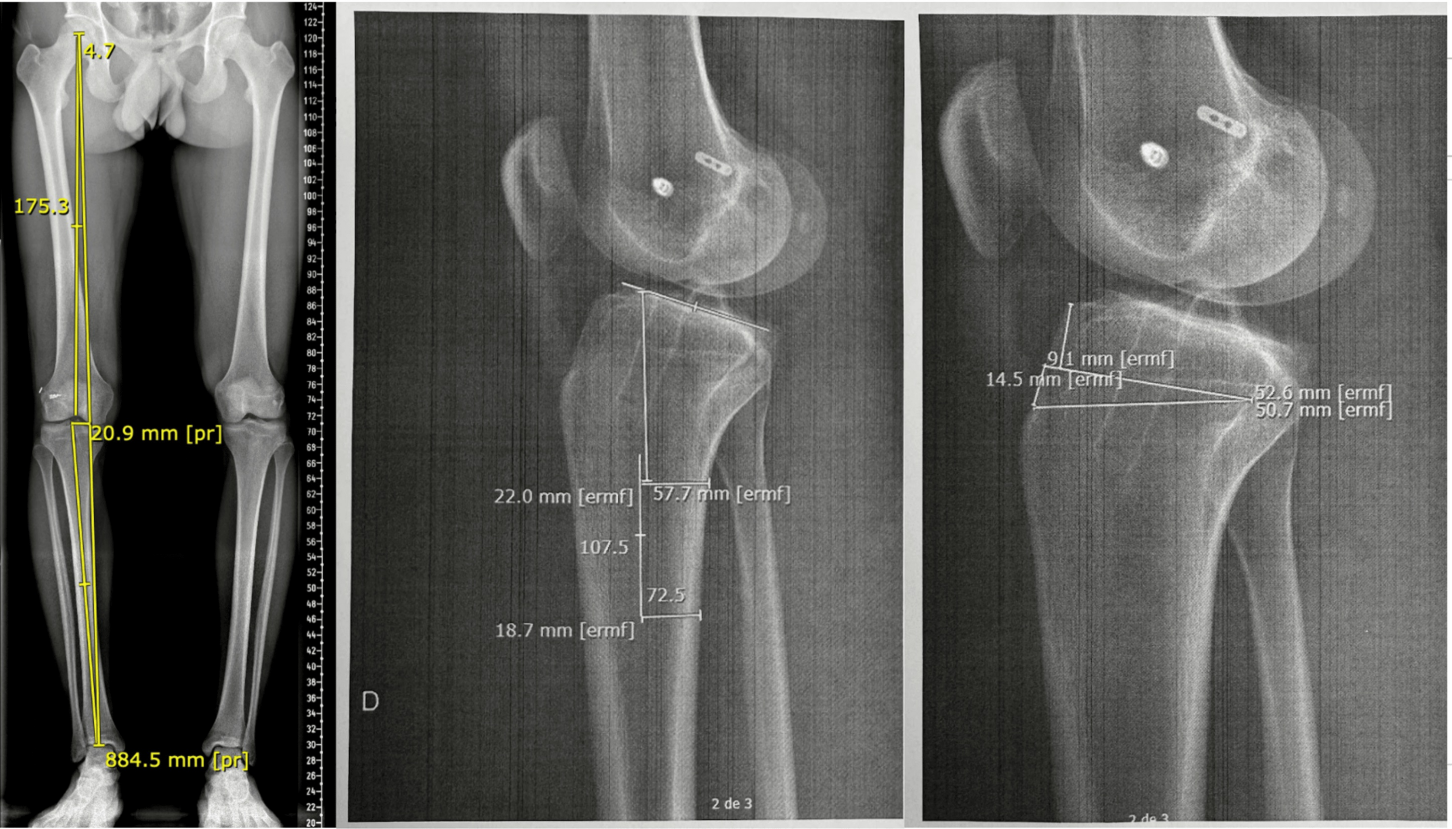
Preoperative planning and posterior tibial slope measurement Left: long-leg standing radiograph demonstrating mild bilateral varus alignment. Center: measurement of posterior tibial slope on lateral radiograph using the medial tibial plateau. Two points along the tibial diaphysis are selected to define the longitudinal tibial axis, and the angle between this axis and the medial tibial plateau is measured, revealing a posterior tibial slope of 17°. Right: preoperative planning of slope correction. A target posterior tibial slope of 7° was established, corresponding to a planned correction of 10°, resulting in a calculated anterior closing-wedge osteotomy height of approximately 9 mm.

A comprehensive revision workup was completed, including long-leg standing radiographs and computed tomography, which demonstrated an increased PTS. Given the presence of graft failure in the setting of pathological PTS, revision ACL reconstruction combined with a slope-reducing proximal tibial osteotomy was indicated (Figure 1).

**Figure 2:**
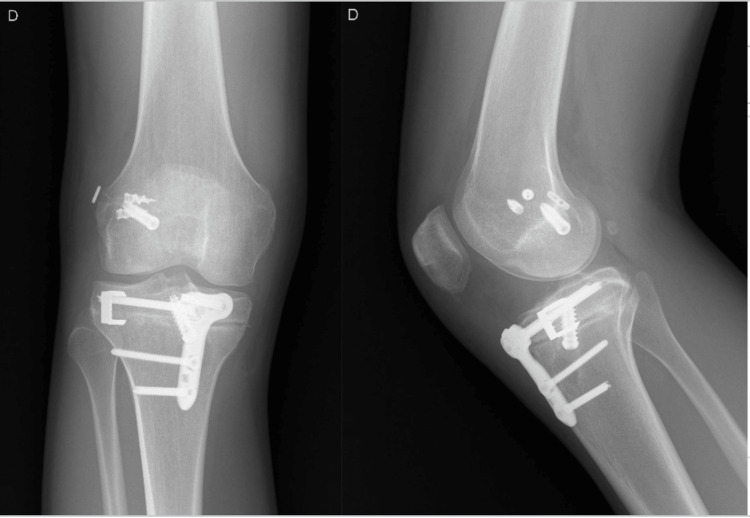
Postoperative radiographic assessment Postoperative lateral radiographs demonstrating the correction of the posterior tibial slope according to the preoperative plan, with the restoration of sagittal alignment following slope-reducing proximal tibial osteotomy.

Preoperative planning focused on sagittal alignment correction, targeting a PTS reduction to restore more physiological knee biomechanics. The patient underwent revision ACL reconstruction associated with an anterior closing-wedge proximal tibial osteotomy (Figure 2).

Postoperatively, the patient demonstrated favorable clinical evolution, with satisfactory functional outcomes and no subsequent episodes of instability or graft re-rupture at follow-up.

Clinical outcomes of slope-reducing osteotomy

Clinical outcome data on slope-reducing osteotomy in revision ACL reconstruction are limited but increasingly supportive. Available series suggest that slope reduction leads to improved knee stability, decreased anterior tibial translation, and reduced rates of recurrent graft failure in appropriately selected patients [7,17].

Mazy et al. demonstrated that even adjunctive procedures such as lateral extra-articular tenodesis may fail to sufficiently protect the graft in the presence of markedly increased PTS, further supporting the rationale for addressing sagittal alignment directly in selected cases [19].

Although high-level comparative studies are lacking, the consistency of biomechanical data and emerging clinical evidence suggest that slope-reducing osteotomy may play an important role in revision ACL reconstruction when excessive PTS is identified as a contributing factor [7,17]. 

Limitations and risks

Slope-reducing osteotomy is a technically demanding procedure and should not be considered a benign adjunct to revision ACL reconstruction. Potential complications include nonunion, hardware-related issues, neurovascular injury, and alteration of knee kinematics. Additionally, inappropriate patient selection or excessive slope correction may negatively affect posterior cruciate ligament function or overall knee biomechanics [7,17].

Given these considerations, slope-reducing osteotomy should be reserved for experienced surgeons and performed in carefully selected patients after thorough preoperative evaluation.

Clinical implications

Slope-reducing osteotomy represents a powerful biomechanical tool in the management of revision ACL reconstruction in selected patients with increased PTS. When appropriately indicated, correction of sagittal alignment may reduce graft loading, improve knee stability, and decrease the risk of recurrent graft failure. However, this technique should be integrated into a comprehensive revision strategy rather than applied routinely.

## Conclusions

Revision ACL reconstruction requires a comprehensive understanding of the multifactorial mechanisms that contribute to graft failure. Among biomechanical factors, increased PTS has emerged as a clinically relevant contributor to excessive anterior tibial translation and increased graft loading, particularly in the revision setting. Current biomechanical, clinical, and consensus-based evidence supports PTS as a potentially modifiable risk factor in selected patients.

Slope-reducing osteotomy should be considered as part of a targeted revision strategy when pathological PTS, most commonly values ≥12°, is identified as a relevant contributor to graft failure. Importantly, this procedure should not be applied routinely, as increased PTS represents a necessary but not sufficient condition for surgical correction. When appropriately selected and integrated into a comprehensive revision approach, slope-reducing osteotomy may improve knee stability and enhance graft survival in revision ACL reconstruction.
